# South African professional Super Rugby players’ lived experiences of career-related traumatic injuries: A descriptive phenomenological analysis

**DOI:** 10.17159/2078-516X/2020/v32i1a8622

**Published:** 2020-01-01

**Authors:** T M Hall, P J Basson, J S Patricios

**Affiliations:** 1 Counselling Psychologist, Private Practice, Sea Point, Western Cape, South Africa; 2 Counselling Psychologist, Lecturer, Department of Psychology, University of Johannesburg, Auckland Park, Johannesburg; 3 Sport and Exercise Medicine Physician, Wits Sport and Health (WISH), Faculty of Health Sciences, University of the Witwatersrand, Johannesburg

**Keywords:** trauma, severe injury, descriptive phenomenology, psychology

## Abstract

**Background:**

Historically, non-career-ending traumatic rugby injury (TRI) has been viewed from a predominantly biological perspective. However, dimensional perspectives, such as the biopsychosocial model, have highlighted the need to incorporate psychosocial understandings of TRI into treatment plans.

**Aim:**

To describe the lived experiences of a cohort of traumatically injured South African Super Rugby players in order to add to the body of literature on the subject of TRI experience.

**Methods:**

The employment of a qualitative, descriptive phenomenological method was used to achieve the research outcome.

**Discussion:**

Common descriptive themes indicated that TRI seems to exist within three stages: the initial, emotional and subsequent reactions to the traumatic injury. Sub-themes described within each stage included attempts at remaining positive and appraising the severity of the injury during onset, fear responses and concomitant feelings of loss related to foregone career opportunities during the emotional reactions stage, the employment of coping mechanisms, and relying on specific support structures during subsequent reactions. Two novel experiences revealed within this study and not reported in the international literature included the injured players’ reliance on compartmentalisation and positive religious belief structures as coping strategies. All themes were reduced to descriptive phenomenological essences that describe a lifeworld or biopsychosocial experience of TRI.

**Conclusion:**

Themes drawn from this study can be applied in the future design and implementation of expanded studies and psychological interventions aimed at assisting traumatically injured rugby players during their recovery process. The identified themes affirm aspects from the international literature while highlighting some uniquely South African outcomes.

In many instances, professional sport is a lucrative livelihood in which athletes are paid commercially competitive salaries. Injuries are costly financially and personally to competitors and their employers, and may impact career longevity. This is particularly relevant to professional sports, such as rugby union (rugby), that require a significant level of physical contact and in which a relatively high number of injuries occur. For example, Schwellnus et al.^[1]^ suggested that per season, 55% of Super Rugby players experience injuries that require more than one week of rest and rehabilitation before they re-enter competition. Hence, evidence-based biopsychosocial injury information that can inform effective recovery protocols is sought after within the rugby environment.

Professional rugby players can be affected physically, psychologically and financially by severe rugby injury (SRI).^[2]^ SRI is defined by Fuller et al.^[3]^ in their consensus statement on injury definitions in rugby union as a career-related injury that requires more than 28 days’ physical recovery.^[3]^ Although, historically speaking, research around the effects of SRI revolved around physical aspects,^[2]^ a description of the psychological components of rugby career-related stressors have also evolved. For example, Nicholls et al.^[6]^ reported that injury risk was the main stressor experienced by professional Heineken Cup rugby players during the 2004 season. More recently, studies aimed at understanding psychosocial sequelae associated with SRI have emerged,^[4,5]^ indicating that these issues can negatively affect both effective physical recovery and playing form.^[2,4]^

Arvinen-Barrow et al.^[2]^ found that the majority of professional rugby players will experience at least one SRI during their careers, and that it is considered a significant stressor. According to these authors, as well as more recent researchers,^[7]^ SRI includes negative psychosocial concomitants that can affect the individual across multiple facets of their lifeworld. Additionally, the results of research aimed at understanding the negative psychosocial consequences of SRI allude to a variety of biopsychosocial experiences that can be considered as traumatic, including the oftentimes stage-wise progression of the traumatic experience.^[8]^ The recognition of an SRI experience as being traumatic could assist in the development of integrated, evidence-based and effective SRI recovery protocols.

A traumatic event includes the ‘exposure to actual or threatened death, serious injury, or sexual violence’.^[9]^ The effect of a traumatic event may incorporate psychological ramifications, such as a sudden sense of helplessness or the loss of control of one’s ability to feel safe or secure within one’s own body and/or the outside world.^[10]^ Psychologically, trauma can be viewed as a situation or an event that shatters a person’s basic assumptions about themselves and the world they live in.^[11]^ For the purposes of this research, an SRI is considered as a traumatic event, which has threatened the anatomical integrity of a competitor. Therefore, a traumatic rugby injury (TRI) is an SRI that consists of its related physical, psychological, social, financial, and career-impacting consequences.^[3,10]^

Research focused on understanding negative psychosocial experiences related to traumatic sports injuries, including SRIs, has begun to emerge primarily outside of South Africa.^[4,5,12,13]^ Studies aimed at understanding the psychosocial consequences of traumatic injuries sustained by athletes have revealed various stages of progression experiences, including an information processing stage, an emotional upheaval stage, and a positive outlook stage of severe injury.^[12]^

## Traumatic rugby injury experiences

A review of the literature from Australia and the United Kingdom revealed certain negative psychosocial factors experienced by traumatically injured professional rugby players. These include negative opinions of the team environment, increased perceptions of pain, frustration, loss of identity within the team, pressure to play while still being injured, and guilt regarding not being able to contribute to the team.^[14]^ Self-doubt and stress (career, medical, financial and social) were suggested as factors associated with TRI by Arvinen-Barrow et al.^[2]^ Evans et al.^[5]^ outlined perceptions related to the following associated with TRI:

1. a loss of independence
2. increased levels of negative social comparison,
3. unwanted attention, loss of routine,
4. feelings of isolation and boredom,
5. anxiety related to incapacitation,
6. medical diagnostic uncertainty and injury prognosis,
7. disruptions in self-image, fears around weight loss,
8. concerns regarding the risk of re-injury,
9. missed competition opportunities,
10. external and internal pressures to recover
11. future performance anxiety

The stigma related to negative perceptions of psychological support regarding both team coaches and administration were factors highlighted by Green et al.^[15]^ Finally, Carson and Polman^[4]^ mentioned that professional rugby players who have experienced a TRI tend to employ a variety of cognitive coping strategies, specifically cognitive avoidance and blocking during TRI recovery.

Outlined psychosocial consequences of career-related TRI, as well as the documented stage-wise progression of the athlete’s injury experience, have been described outside of South Africa. The aim of this research was to define the lived experiences of traumatically injured South African Super Rugby players.

## Methods

### Study design

To achieve the aims of this study, the selected qualitative research lens was descriptive phenomenology. Descriptive phenomenology aims to provide comprehensive information around specific lived experiences and reduce that information to a description of its essence.^[16]^ A phenomenological essence is what would be uncovered if all layers of description are stripped away and the phenomenon under investigation reveals itself as it would if it could.^[16]^

The phenomenon under investigation in this study is the experience of TRI. Specific participant biographical and injury information can be relevant in an interpretation of the descriptive themes generated from the research. However, this information is not specifically pertinent to the descriptive phenomenological method. Simply put, descriptive essences of professional, career-related TRI exist within the experience of the phenomenon regardless of specific pre-existing injury information

Finally, although qualitative phenomenological findings are not universally generalisable beyond the research sample, significant essences that are revealed are seen as indications of shared human experiences of the phenomenon under investigation.^[16]^

### Participants

Three research participants were selected for this descriptive analysis via purposive sampling. The sample size was in accordance with Giorgi’s^[17]^ methodological recommendations. Participants were South African professional Super Rugby players contracted by the same franchise to participate in the 2016 Super Rugby competition and listed as A, B, and C. All three participants were between the ages of 24 and 30 years old, considered old enough to be established within the profession but young enough to expect to continue to play after TRI recovery.

All three participants were recovering from a TRI, as defined by Fuller et al.^[3]^ and had undergone surgery as part of their recovery protocol. Differences in TRI which included type, timing, prognosis and number of previous injuries could have affected the participant-specific findings of this study. However, as this phenomenological study aimed to extract descriptive universal essences of the phenomenon of career-related TRI (i.e. essences of TRI) specific participant injury information was not relevant to this aim.

The descriptive phenomenological method relies on the participants’ ability to articulate their experiences of the phenomenon. All participants could adequately communicate in the English language, the first language of the researcher.

### Recruitment

Ethical clearance to conduct this study was obtained from the University of Johannesburg’s Human Research Ethics Committee. A professional franchise was approached via the team’s sports physician in order to assist in the recruitment of the research participants. An invitation letter to participate in this research was sent to each potential participant. Information covering the objectives of the study, the interview process, and any potential negative outcomes were provided. Each participant gave their written informed consent.

### Data collection

Data were collected via three open-ended interviews that were conducted in a conversational (informal) style. The open-ended interview technique involves the interviewer posing a broad, non-descriptive and non-definitive question regarding the phenomenon under review.^[16]^ The same open-ended question posed to each study participant was, ‘Please could you describe what you are experiencing as a result of your injury?’. All three interviews were recorded on an audio recorder. The recordings were then transcribed verbatim.

### Descriptive phenomenological analysis

Descriptive phenomenological research requires that all researcher preconceptions be suspended or bracketed, as they may contaminate interpretations of the participants’ interviews. Giorgi’s^[17]^ steps for descriptive phenomenological analysis were employed in the analysis of the transcribed interviews. These steps consisted of the following. 1) Each transcribed interview was read as a whole in order to gain a global, holistic understanding of the data.^[17]^ 2) A second reading of the description was undertaken, while experiences of meaning transitions were noted (still in the words of each participant) in order to reduce the descriptions into meaning units. 3) The researcher then transformed the meaning units into descriptions that contained explicit psychological information of importance and value connected to the phenomenon under investigation.^[17]^ 4) The psychological descriptions of the phenomenon under review were reduced further by re-examining and re-organising them in order to come to a synthesis of psychologically-based meaning units.^[17]^ 5) Appropriate descriptions of individual experiences were compared and integrated across participants in order to elicit potential commonalities and arrive at a description of general experiences. 6) Themes common to at least two of the three interviewees were re-examined, re-organised, and synthesised into overarching descriptive universal essences found in the participants’ experiences.^[17]^

## Discussion

Three general experiences of career-related TRI comprised of initial, emotional, and subsequent reactions to the traumatic injury were revealed. These findings were described as a stagewise progression of TRI experience. Findings were aligned with previously described stages of athletic injury rehabilitation, such as 1) the information processing stage, 2) the emotional upheaval stage, and 3) the positive outlook stage.^[12]^

Initial reactions to the traumatic injury comprised the participants’ attempts at positivity and their cognitive appraisals of the injury’s severity. Emotional reactions to the traumatic injury included participants’ descriptions of confusion around diagnoses, fear reactions (such as fear of surgical procedures and fear of financial losses), and feelings of loss (such as loss of career opportunities, physical fitness, and team connection). Finally, subsequent reactions to the traumatic injury involved participants describing their awareness and implementation of adaptive coping mechanisms, including goal directedness, adaptive avoidance, compartmentalisation, visualisations, positive religious belief structures, and the reliance on insider, outsider, and medical team support structures.

Although three distinct stages of TRI experience were described in this text, one could reasonably argue that emotional components exist across all three stages of experience. This aligns with the biopsychosocial model of understanding. However, the initial reactions to TRI, as well as the subsequent reactions, were described in terms of purposeful attempts to control experiences rather than spontaneous emotional experiences.

### Initial reactions to the traumatic injury

#### Attempts at positivity

All participants described a degree of positivity in reaction to the shock of the onset of their injury. Mention was made of consciously employing positivity as an initial reaction in order to defend against future-orientated fears. One participant described positivity as having arisen due to his lack of injury experience, his trust in the medical team, and the positive responses of his team mates.

*A lot of guys have … ops [surgery] done … and all of them, like everyone, is positive …’… you going to be alright, don’t stress, you’re going to do this and this with, Doc … you’ll start your rehab in so many weeks and whatever’*

This affirms the observation of Kruger et al.^[18]^ who noted positivity, together with cognitive self-appraisals, as one of the possible psychological skills that South African rugby players competing at transnational levels appear to have at their disposal.

#### Cognitive appraisals of injury severity

Each participant described the attempts made to assess the severity of their injuries. One participant indicated that he tried to rationalise his injury as part of his endeavours to assess the severity thereof.

*… there wasn’t a lot of emotion [during his reactions to the traumatic injury] but a lot of trying to rationalise things … I told myself, ‘you know what? it’s [traumatic SRI] already happened, where to from here?’… and then … my mind was starting to think like, ‘… sherbet [expression of frustration], I hope this [SRI] isn’t bad. I can feel it’*.

Concannon and Pringle^[12]^ found in their study that during the initial or information processing stage of the injury, athletes may engage in self-reflection as a way of processing information around the injury, including the extent of physical damage done and the likelihood of returning to competition.

### Emotional reactions to the traumatic injury

#### Diagnostic confusion

All three participants described an experience of feeling confused related to either misdiagnoses or miscommunication around the original diagnosis. This feeling led to participants describing varying degrees of emotional destabilisation ranging from feeling disappointed to feeling angry.

*… and all of them [doctors across provincial and national teams] have different opinions of when I’ll be ready, or what was wrong initially, or what is wrong now … it’s challenging having a lot of people speak into your life at the same time about one thing but saying different things*.

The experience of confusion and its concomitant challenges has been documented by Evans et al.^[5]^ in their research aimed at understanding stressors experienced by injured athletes. These authors suggested that injured athletes experience stress and worry related to confusing injury diagnostic information.

#### Fear reactions

Participants described various fear reactions in response to their traumatic SRI which was similar to that found in the literature related to injured elite athletes across several sporting codes.^[13]^

#### Fear around a surgical procedure

Participants described experiencing fear around the need for a surgical procedure. One individual expressed feeling ‘nervous’ prior to undergoing an exploratory operation.

*Look, going into the [surgical] operation, I was still nervous*.

#### Fear of financial loss

Participants expressed fear regarding the potential financial losses due to their TRIs. One participant mentioned that the potential loss of a future contract would place him in a difficult financial situation.

*What’s going to happen to my finances? … I think that might be where the fear is coming from … if that contract is taken away from you [me] or you [I’m] not able to play rugby in the next year … I think that’s where the fear would probably creep in*.

Both Evans et al.^[5]^ and Arvinen-Barrow et al.^[2]^ suggested that injured professional rugby players’ feelings of stress often include their perceptions of not being able to cope with financial demands placed on them.

#### Feelings of loss

Interviewees described painful emotional reactions to the realisation of what they had lost in terms of ‘near future’ career opportunities. One such reaction included the following:

*I had aspirations to play on the end of year tour, last year … and then this was like my goal for the year (crying). Ja, (sigh) … I got the opportunity and everything and … missed it this year. I feel like it’s not that I let the opportunity go or whatever but I felt like the opportunity is gone*.

Evans et al.^[5]^ suggested that injured professional rugby players experience stress associated with their realisation of missed career opportunities across the injury onset and the rehabilitation stages of the injury process.

### Subsequent reactions to the traumatic injury

#### Adaptive coping mechanisms

All three participants described the employment of adaptive coping mechanisms during this stage of their injury trajectory. Coping mechanisms that were mentioned by the participants included goal directedness, adaptive avoidance, compartmentalisation, visualisations, and using positive religious belief structures. Carson and Polman^[19]^ mentioned that elite professional rugby players tend knowingly to employ coping strategies, mechanisms and skills, aimed at managing stressful demands - including traumatic injuries.

#### Goal directedness

Participants described directing their consciousness towards using specific recovery and rehabilitation goals, as well as general career goals. One participant mentioned directing his attention to his goal of returning to play rugby, while activities not aligned to that goal were set aside.

*… You just keep going and the main goal [playing rugby again] remains the main goal … Anything that’s not preferable [aligned to recovery] can wait. Everything should be focused on me getting back [to competitive play]*.

Carson and Polman^[4]^ suggested that effective goal-setting strategies seemed to assist injured professional rugby players in their attempts to return to competitive play.

#### Adaptive avoidance

Interviewees described intentionally avoiding those individuals or groups they thought would distract them from their recovery goals. Mention was made of managing fears related to being negatively influenced by family members by avoiding them until a later stage of recovery.

… *They [*his family*] might pull my energy in a different direction [*from his goal of recovery*] and I start to slack in terms of what I’ve set out to do for myself (sigh) … that’s a sacrifice I had to make*.

Adaptive avoidance (including cognitive avoidance and blocking) has been described in the literature by authors such as Carson and Polman^[4]^ as one such coping mechanism employed by professional rugby players.

#### Compartmentalisation

Participants described compartmentalising their daily activities during their recovery. They mentioned how the separation of rugby and rehabilitation tasks from other life interests assisted them in coping with their SRI recovery.

*Rugby is not my life, it’s part of my life … I can go home to my family and, consciously, I disconnect from being a professional rugby player … I’m a father and a husband and through that, through that disconnection, I have peace*.

Specific literature pertaining to the employment of compartmentalisation as a coping mechanism by injured sports people was not found. This finding could also suggest a novel and South African component of TRI recovery.

#### Visualisations

Participants mentioned the use of positive visualisation techniques to control their thoughts and emotions during their recovery processes. One individual described how visualisation techniques ‘triggered’ him into remaining motivated to play rugby again.

*I believe that visualising playing in that jersey [team rugby jersey] again really helps me … it’s almost like a trigger to see it [playing again] … it’s easy to say its motivation. It is to an extent but it triggers my mind*.

Carson and Polman^[19]^ maintained that use of imagery as a coping strategy has proved to be beneficial in injured rugby players’ recovery processes.

#### Positive religious belief structures

A reliance on religious belief structures in order to cope with a traumatic rugby injury was described by two participants. One participant mentioned how his practices of faith and prayer assisted him in his belief that he would be able to control the outcomes of his recovery process.

*I’d go to church and the people would pray for me and it will be the same thing, just speak it [positive recovery] into existence and now I’m sitting here and I’m thinking, ‘flip [colloquial expression used in this case as surprise], I could be back [playing rugby] in six months (laughing)’*.

No existing literature pertaining to the employment of positive religious belief structures as a coping mechanism regarding injured athletes or rugby players was found. Hence, this coping mechanism could be considered a South African component of psychosocial TRI recovery, especially if replicated in additional studies across similar contexts.

### Support structures

Participants described their awareness of how certain support structures, including both insider, outsider, and medical team support, positively assisted them during their respective rehabilitation.

Williams and Appaneal^[13]^ suggested that injured athletes are often influenced, either negatively or positively, by their support structures and that those injured athletes who perceive a lack of social support might experience negative cognitive perceptions in their self-worth and self-confidence.

#### Insider support

All participants described support received from teammates as being beneficial. One individual communicated how he felt supported by other injured teammates.

*… having guys that are also injured with you [me] … they also have niggles, they also come there, with Doc [biokineticist] and [we] do our stuff together. Even though we’re doing completely different things, it’s just lekker [slang expression for ‘pleasant’] having guys that [who] are in a similar scenario as you [me] … having them around was nice*.

Carson and Polman^[19]^ suggested that injured professional Rugby Union players might become motivated to recover via the exposure to positive team support structures.

#### Outsider support

Outsider support was described by interviewees as being helpful to them during their convalescence. One interviewee mentioned his girlfriend as being particularly supportive of him at both physical and emotional levels.

*My [girlfriend] was also very supportive … I think she wants to try help. She did help me a lot … also, [I] not knowing what to expect [from the surgical procedure], neither did she*.

Williams and Appaneal^[13]^ suggested that the availability of social support in the form of relationships both with teammates and family is vital regarding the ability of injured athletes’ to protect themselves from feelings of neglect and abandonment.

#### Medical team support

Participants described themselves as reacting positively to the support that they received from their medical team during the rehabilitation stages of their injury. One participant mentioned the following:

*He [the biokineticist] just answered all my questions … he’ll be [he was] straight with me … he’ll say my external rotation is looking good [or] he tells me, ‘okay, that’s not good, we can work on that’ … he [the biokineticist] was also just honest with me. He didn’t sugar coat anything*.

In their 2017 study, Carson and Polman^[4]^ proposed that an injured professional rugby player’s recovery seemed to be assisted by the support received from the medical team treating him.

### Universal essences

Common phenomenological essences were divulged by all three participants which included the following: being confronted with both physical and psychological limitations, while intentionally attempting to make meaning out of the TRI experience and the recovery process.

All three participants attempted to create meaning during their recovery process via the intentional recognition of their physical injury, the experience of emotional reactions, and the deliberate and directional implementation of coping strategies which included the seeking out of social support.

### Limitations

As with most qualitative research, results should not be generalised across all rugby playing populations due to small sample sizes, such as in this research study. Furthermore, the descriptive phenomenological method does not aim to interpret findings. Hence, enquiries regarding how or why descriptions of the TRI experience exist were limited in this research.

## Conclusion

The occurrence of TRI within a sample of professional South African Super Rugby players revealed a stagewise biopsychosocial experience. This experience was reduced to universal essences, which included a reliance on and confrontation with both physical and psychological limitations, while intentionally striving towards establishing their significance. As previously discussed, no traumatic event occurs entirely from the physical, psychological, or social point of view. Rather, there is an interaction between the three components of the experience. According to this research, there appear to be additional factors unique to the South African experience of TRI.

The two descriptive phenomenological themes in this study and not found in the international literature are coping strategies of *compartmentalisation* and the reliance on *positive religious belief structures*. The fact that two themes, possibly unique to the South African Super Rugby population, were extracted from this study adds credibility to the idea that the experience of career-related TRI is embedded in the biopsychosocial lifeworld of the participant.

Further investigations regarding how unique experiences of TRI can affect recovery protocols across different and larger cohorts will add further value to the subject. Furthermore, quantitative studies aimed at understanding what specific experiences positively or negatively influence recovery protocols could assist in the development of specific but malleable psychosocial interventions aimed at TRI recovery.

## References

[1] Schwellnus MP Thomson A Derman W More than 50% of players sustained a time-loss injury (>1 day of lost training or playing time) during the 2012 Super Rugby Union Tournament: a prospective cohort study of 17 340 player-hours Br J Sports Med 2014 48 17 1306 1315 10.1136/bjsports-2014-093745 24982503

[2] Arvinen-Barrow M Massey WV Hemmings B Role of sport medicine professionals in addressing psychosocial aspects of sport-injury rehabilitation: Professional athletes’ views J Athl Train 2014 49 6 764 772 10.4085/1062-6050-49.3.44 25243737 PMC4264648

[3] Fuller CW Molloy MG Bagate C Consensus statement on injury definitions and data collection procedures for studies of injuries in rugby union Br J Sports Med 2007 41 5 328 331 10.1136/bjsm.2006.033282 17452684 PMC2659070

[4] Carson F Polman RC Self-determined motivation in rehabilitating professional rugby union players BMC Sports Sci Med Rehabil 2017 9 2 10.1186/s13102-016-0065-6 28116102 PMC5241994

[5] Evans L Wadey R Hanton S Stressors experienced by injured athletes J Sports Sci 2012 30 9 917 927 10.1080/02640414.2012.682078 22551525

[6] Nicholls AR Holt NL Polman RC Stressors, coping, and coping effectiveness among professional rugby union players Sport Psychol 2006 20 3 314 329 10.1123/tsp.20.3.314

[7] Hurley OA Impact of player injuries on teams' mental states, and subsequent performances, at the Rugby World Cup 2015 Front Psychol 2016 7 807 10.3389/fpsyg.2016.00807 27375511 PMC4891352

[8] Eagle G An integrative model for brief term intervention in the treatment of psychological trauma Int J Psycother 1998 3 2 135 146 Available from [https://search.proquest.com/openview/a23dfb9c1b6943289b2eb95a4bdb3b80/1?pq-origsite=gscholar&cbl=32791

[9] American Psychiatric Association Diagnostic and statistical manual of mental disorders s.l American Psychiatric Publishing 5th ed 2013 271

[10] Corbett L Milton M Existential therapy: A useful approach to trauma? Couns Psychol Rev 2011 26 1 62 74 Retrieved from: https://psycnet.apa.org/record/2011-06757-006

[11] Janoff-Bulman R Assumptive worlds and the stress of traumatic events: Applications of the schema construct Soc Cogn 1989 7 2 113 136 10.1521/soco.1989.7.2.113

[12] Concannon M Pringle B Psychology in sports injury rehabilitation Br J Nurs 2012 21 8 484 490 10.12968/bjon.2012.21.8.484 22585077

[13] Williams RA Appaneal RN Social support and sport injury Int J Athl Ther Train 2010 15 4 46 49 10.1123/att.15.4.46

[14] Cresswell SL Eklund RC The nature of player burnout in rugby: Key characteristics and attributions J Appl Sport Psychol 2006 18 3 219 239 10.1080/10413200600830299

[15] Green M Morgan G Manley AJ Elite rugby league players’ attitudes towards sport psychology consulting SEPR 2012 8 1 32 45 10.1037/spy0000103

[16] Lindegger G Research methods in clinical research Terre Blanche M Durrheim K Painter D Research in practice: Applied methods for the social sciences 2nd rev ed Cape Town UCT Press 2006 455 475

[17] Giorgi A The descriptive phenomenological psychological method J Phenomenol Psychol 2012 43 1 3 12 10.1163/156916212X632934

[18] Kruger P Potgieter J Malan D Prior experience, cognitive perceptions and psychosocial skills of senior South African rugby players S Afr J Res Sport Phys Educ 2010 32 1 69 84 10.4314/sajrs.v32i1.54101

[19] Carson F Polman RC The facilitative nature of avoidance coping within sports injury rehabilitation Scand J Med Sci Sports 2010 20 2 235 240 10.1111/j.1600-0838.2009.00890.x 19422659

